# Early detection of osteoarthritis in rabbits using MRI with a double-contrast agent

**DOI:** 10.1186/s12891-018-2002-1

**Published:** 2018-03-13

**Authors:** Okihiro Onishi, Kazuya Ikoma, Masamitsu Kido, Yukichi Kabuto, Keiichiro Ueshima, Ken-ichi Matsuda, Masaki Tanaka, Toshikazu Kubo

**Affiliations:** 1 Department of Orthopaedics, Graduate School of Medical Science, Kyoto Prefectural University of Medicine, 602-8566 465, Kajiicho, Kamigyo-ku Kyoto-shi, Kyoto, Japan; 2 Department of Anatomy and Neurobiology, Graduate School of Medical Science, Kyoto Prefectural University of Medicine, Kyoto, Japan

**Keywords:** Contrast agent, Rabbit model, Osteoarthritis

## Abstract

**Background:**

Articular cartilage degeneration has been evaluated by magnetic resonance imaging (MRI). However, this method has several problems, including its time-consuming nature and the requirement of a high magnetic field or specialized hardware. The purpose of this study was to sequentially assess early degenerative changes in rabbit knee articular cartilage using MRI with a new double-contrast agent.

**Methods:**

We induced osteoarthritis (OA) in the right knee of rabbits by anterior cruciate ligament transection and partial medial meniscectomy. Proton density-weighted images and T_2_-calculated images were obtained before and after contrast agent injection into the knee. The signal intensity ratio (SIR) values on the proton density-weighted images were calculated by dividing the signal intensity of the articular cartilage by that of joint fluid. Six rabbits were examined using MRI at 2 (designated 2-w OA) and 4 weeks (4-w OA) after the operation. Histological examination was performed 4 weeks after the operation. One rabbit was histologically examined 2 weeks after the operation. The control consisted of six rabbits that were not subjected to the operation. The SIR values, T_2_ values and the thicknesses of the cartilage of the 2-w OA, 4-w OA and the control before and after contrast agent injection were analyzed. The Mankin score and OARSI (Osteoarthritis Research Society International) score were used for the histological evaluation.

**Results:**

Significant differences in the SIR and T_2_ values of the medial and lateral condyles of the femur were found between the control and the 4-w OA only after contrast agent injection. No significant differences were found in the SIR and T_2_ values before contrast agent injection between the control, the 2-w OA and 4-w OA. The thickness of the articular cartilage revealed no significant differences. In the histological assessment, the Mankin score and OARSI score sequentially increased from the control to the 4-w OA.

**Conclusion:**

We evaluated the SIR and T_2_ values of the knees in a rabbit OA model and a control model using a new double-contrast agent. MRI with this agent enabled OA detection earlier than using conventional MRI.

## Background

Osteoarthritis (OA) leads to degeneration and wear of the articular cartilage, sclerosis of the subchondral bone, and synovitis, causing joint pain, limitation of joint movement, and muscular atrophy [1]. Many older individuals suffer from OA, which reduces their quality of life and increases the mortality rate [2, 3]. The progression of OA requires painkillers and surgery, such as prosthetic arthroplasty, osteotomy, and arthroscopic operations. Early diagnosis and treatment of OA would not only prevent the development of more severe OA in many patients but would also decrease social costs, including medical expenses and human resources.

Exercise therapy, orthosis, and oral administration of non-steroid anti-inflammatory drugs, opioids, and acetaminophen are used to treat early-stage OA, and the effects of these agents are being examined. However, a method for early detection of articular cartilage degeneration has not been established [4]. The Kellgren and Lawrence scale is clinically used to evaluate the grade of knee OA [5]; however, this scale evaluates the grade of OA based on articular cartilage wear and is therefore not applicable for evaluating early-stage OA, in which the articular cartilage thickness has not decreased [6]. Computed tomography exposes patients to high radiation doses and is therefore not an ideal choice for evaluating OA [7, 8].

Recently, the use of magnetic resonance imaging (MRI) has been evaluated to determine the quality of articular cartilage. OA reduces proteoglycan (PG) and collagen content in articular cartilage and increases the content of water molecules. Qualitative evaluations of articular cartilage using MRI can be divided into two types. One type mainly detects hydration and reductions in collagen density in articular cartilage, whereas the other type mainly detects decreases in PG in articular cartilage. The former includes T_2_ mapping and diffusion-weighted imaging, whereas the latter includes delayed gadolinium-enhanced MRI (dGEMRIC), T_1ρ_ mapping, glycosaminoglycan chemical exchange saturation transfer (gag CEST), and ^23^Na MRI [9–11]. Recht et al. reported that T_2_-weighted images showed articular cartilage of the human knee, although thin-layered cartilage, such as that in the hip joint and ankle joint, is difficult to depict clearly [12]. It is difficult to use T_1ρ_-calculated images because their acquisition requires a long period of time, and the reference values cannot be set because T_1ρ_ values vary with the magnetic field of the MRI device [13]. dGEMRIC is used to detect degenerative changes in articular cartilage; however, problems with this technique include a long acquisition time and restrictions for use in patients with renal impairment [14, 15]. Gag CEST requires an MRI device with a high magnetic field [16–18]. ^23^Na MRI requires specialized hardware; therefore, it is difficult to obtain these images in many facilities [19].

Manganese ions shorten T_2_ relaxation times and have been used as components of contrast agents in MRI studies examining the brain and digestive organs [20–22]. In a previous study, the selective visualization of articular cartilage using MRI with a new double-contrast agent containing manganese ions as a positive contrast agent and ferrous and copper ions as negative contrast agents revealed thin-layered structures that were difficult to visualize using conventional methods [23]. Accordingly, rabbit knee cartilage was successfully and vividly delineated ex vivo in that study. As articular cartilage degenerates, its T_2_ relaxation time of articular cartilage becomes longer. We hypothesized that selective visualization of articular cartilage using MRI could be used to detect early degenerative changes in articular cartilage. The purpose of this study was to sequentially assess degenerative changes in rabbit knee articular cartilage using MRI with a new double-contrast agent and compare the results with observations obtained using histological methods.

## Methods

### Animals

This study was conducted in compliance with the guidelines in the Guide for the Care and Use of Laboratory Animals published by the National Institutes of Health and was approved by the Ethics Committee on Animal Experimentation of our institution. Thirteen male Japanese white rabbits (13 to 15-week-old; weighing 3.0 to 3.2 kg; Shimizu Laboratory Supplies Co., Ltd. Kyoto, Japan) were housed in separate cages at the Animal Center of our institution’s animal facility. The rabbits were anesthetized by the inhalation of isoflurane at a rate of 2.5 L/min. OA was induced in the right knee joint by anterior cruciate ligament transection and a partial medial meniscectomy, and the left knee remained intact. Seven rabbits underwent the operation. Six rabbits were examined using MRI at 2 (designated 2-w OA) and 4 weeks (4-w OA) after the operation. Histological examination was performed 4 weeks after the operation. One rabbit was histologically examined 2 weeks after the operation. The control consisted of six rabbits that were not subjected to the operation.

### MRI protocol

**Fig. 1:**
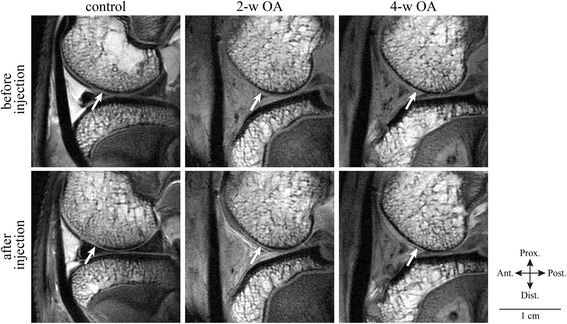
MR images from the present study. Representative proton density-weighted images are shown. The top row shows MR images of the control, the 2-w OA and 4-w OA before contrast agent injection, and the bottom row after contrast agent injection. The arrows indicate the surface of the cartilage in the control, 2-w OA and 4-w OA. Scale bar, 1 cm. Ant. = anterior, Post. = posterior, Prox. = proximal, and Dist. = distal

MRI was performed on a large-bore gradient coil (inside diameter, 210 mm) MRI unit for animals (Varian MRI System 7.04 Tesla; Agilent Technologies, Palo Alto, CA) using a transmitter/receiver surface coil (4 × 3-cm diameter). The rabbits were placed in a supine position, and the knee joint was placed in 90° flexion under the surface coil on the cradle. Each rabbit was anesthetized by the inhalation of isoflurane at a rate of 1.5 L/min while performing MRI. Sagittal images through the medial and lateral condyle of the right femur in a supine position of the 2-w OA and 4-w OA rabbits and the control rabbits were obtained at 2 and 4 weeks postoperatively. MR images were obtained before and 5 min after contrast agent injection (1.0 mL) into the knee joints. A microelement preparation of a high-caloric infusion preparation (Mineric^®^ Nipro Pharma Corp., Osaka, Japan) was used as the contrast agent [23]. Mineric^®^ contains 0.5 mmol/L MnCl_2_, 2.5 mmol/L CuSO_4_, and 17.5 mmol/L FeCl_3_. To evaluate the efficacy of the contrast agent in vivo, proton density-weighted images were obtained before and 5 min after contrast agent injection (Fig. 1). The parameters were as follows: spin-echo sequence; TR, 2000 ms; TE, 11.55 ms; flip angle, 20 degrees; 10 sagittal slices; slice thickness, 1 mm; matrix, 512 × 512 after zero-filling; acquisition time = 8 min 36 s. The signal intensity of the articular cartilage and joint fluid were measured, and the signal intensity ratio (SIR), which was obtained by dividing the signal intensity of the articular cartilage by that of joint fluid, was calculated.

T_2_-calculated images were obtained from fast spin-echo images with 8 TEs. The parameters were as follows: TR, 2000 ms; 8 TEs, 11.55–92.4 ms; echo train length, 2; echo spacing, 11.55; field of view, 40 × 40 mm; 10 sagittal slices; slice thickness, 1 mm; matrix, 256 × 256; acquisition time = 34 min 12 s. Regions of interest (ROI) were selected that corresponded to the weight-bearing areas in the medial and lateral condyle of the femur and the fluid near the area on the sagittal slices. The sagittal slices were obtained perpendicular to the line through the medial and lateral condyle of the femur on the axial slice and parallel to the bone axis of the tibia on the coronal slice. The proton density-weighted images were then referenced when the ROI was set. The MRI findings were reviewed by two orthopedic surgeons.

The thickness of the articular cartilage was measured in proton density-weighted images after contrast agent injection as a conventional assessment of osteoarthritis.

The SIR and T_2_ values of 6 knees of each group were compared.

### Statistical analysis

The SIR values and T_2_ values of the articular cartilage of the medial and lateral condyles of the femur before and after injection of the agent were compared between the control and the 2-w OA, as well as between the control and the 4-w OA by the Mann-Whitney U test. They were compared between the 2-w OA and the 4-w OA by the Wilcoxon signed-rank test. A significant difference was defined as a Bonferroni-corrected *P*-value less than 0.017. The thickness of the articular cartilage of the medial and lateral condyle after injection of the agent was compared between the control and the 2-w OA, as well as between the control and the 4-w OA by the Mann-Whitney U test, and was compared between the 2-w OA and the 4-w OA by the Wilcoxon signed-rank test. A significant difference was defined as a Bonferroni-corrected *P*-value less than 0.017. We assessed variations in the SIR and T_2_ values before injection to after injection by the Wilcoxon signed-rank test. A significant difference was defined as a *P*-value less than 0.05. SPSS ver. 22 (IBM Corp., Armonk, NY, USA) was used for the analysis.

### Histology

One rabbit from the 2-w OA, the four evaluable rabbits from the 4-w OA and one rabbit from the control were assessed. The rabbits were sacrificed by the inhalation of isoflurane at a rate of 2.5 L/min and the intravenous injection of 4.0 mL of pentobarbital sodium (Somnopentyl^®^, Kyoritsuseiyaku Corp., Tokyo, Japan); each rabbit’s knee joint was then dissected. The knee joint was preserved in 10% neutral-buffered formalin solution and decalcified in 10% ethylenediaminetetraacetic acid solution. The tissues were embedded in paraffin and cut at the location in the same manner as the MR images were selected. The tissues were stained with hematoxylin and eosin (H&E) and Safranin O according to the previous study [23]. Histological evaluation of OA was performed using the Mankin score and OARSI (Osteoarthritis Research Society International) score of articular cartilage changes in three slices per rabbit [24, 25]. The averages of the scores of the 2-w OA, 4-w OA and the control were treated as the overall scores of the groups.

## Results

### Comparison of SIRs

**Fig. 2:**
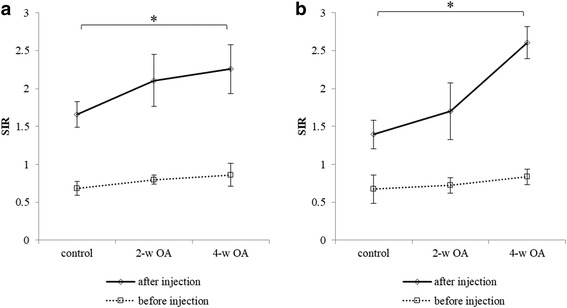
SIR values in proton density-weighted images. The SIR values of the medial condyle of the femur before and after contrast agent injection (**a**) and those of the lateral condyle of the femur before and contrast agent injection (**b**) are shown. * indicates *P* < 0.017, which was defined as a significant change

Significant differences were observed in the SIR values of the medial condyle (*P* = 0.004) and the lateral condyle (*P* = 0.002) only after contrast agent injection between the control and the 4-w OA (Fig. 2). On the contrary, no significant differences were noted in the SIR values of the medial and lateral condyles of the femur before contrast agent injection between the control, 2-w OA, and 4-w OA (Fig. 2). The SIR values after contrast agent injection were significantly greater than those before contrast agent injection (Table 1).

**Table 1:** The comparison of the SIR values before and after contrast agent injection

	Before injection	After injection	
	SIR (SI of cartilage (× 10^4^) / SI of fluid (× 10^4^))	SIR (SI of cartilage (× 10^4^) / SI of fluid (× 10^4^))	*P*-value
(a)			
Control	0.683 ± 0.092 (0.921 ± 0.171 / 1.360 ± 0.268)	1.660 ± 0.169 (1.102 ± 0.188 / 0.680 ± 0.193)	0.028
2-w OA	0.797 ± 0.061 (1.125 ± 0.200 / 1.416 ± 0.247)	2.106 ± 0.344 (1.176 ± 0.306 / 0.573 ± 0.179)	0.028
4-w OA	0.863 ± 0.150 (1.167 ± 0.088 / 1.377 ± 0.179)	2.258 ± 0.322 (1.437 ± 0.324 / 0.641 ± 0.150)	0.028
(b)			
Control	0.674 ± 0.186 (1.016 ± 0.219 / 1.546 ± 0.270)	1.396 ± 0.187 (1.200 ± 0.251 / 0.865 ± 0.167)	0.028
2-w OA	0.723 ± 0.104 (1.059 ± 0.234 / 1.464 ± 0.261)	1.700 ± 0.374 (1.236 ± 0.376 / 0.729 ± 0.158)	0.028
4-w OA	0.835 ± 0.104 (1.196 ± 0.216 / 1.438 ± 0.229)	2.605 ± 0.212 (1.363 ± 0.350 / 0.525 ± 0.141)	0.028

(a) medial condyle, (b) lateral condyle

### Comparison of T_2_ values

**Fig. 3:**
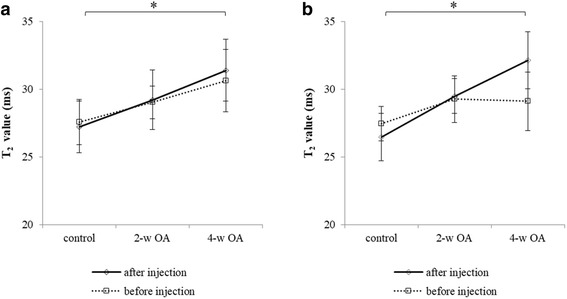
T_2_ values of articular cartilage. The T_2_ values of the medial condyle of the femur before and after contrast agent injection (**a**) and those of the lateral condyle of the femur before and contrast agent injection (**b**) are shown. * indicates *P* < 0.017, which was defined as a significant change

Significant differences in the SIR values of the medial condyle (*P* = 0.009) and the lateral condyle (*P* = 0.002) were observed only after contrast agent injection between the control and 4-w OA (Fig. 3). On the contrary, no significant differences were noted in the T_2_ values of the medial and lateral condyles of the femur before contrast agent injection between the control, 2-w OA and 4-w OA (Fig. 3). The T_2_ values of the lateral condyle in the 4-w OA were significantly greater after contrast agent injection than those prior to injection (Table 2).

**Table 2:** T_2_ Values before and after contrast agent injection

Medial condyle	Before injection	After injection	*P*-value	Lateral condyle	Before injection	After injection	*P*-value
Control	27.6 ± 1.67	27.2 ± 1.92	0.753	Control	27.4 ± 1.25	26.5 ± 1.73	0.116
2-w OA	29.0 ± 1.20	29.2 ± 2.18	0.753	2-w OA	29.3 ± 1.73	29.5 ± 1.29	0.600
4-w OA	30.6 ± 2.29	31.4 ± 2.27	0.249	4-w OA	29.1 ± 2.16	32.1 ± 2.09	0.028

### Comparison of the thickness of the articular cartilage on MRI

**Fig. 4:**
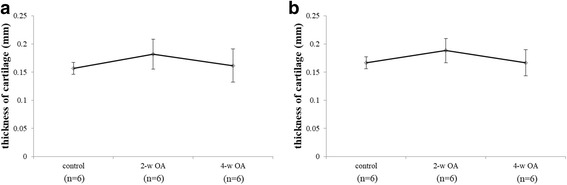
The thickness of articular cartilage in proton density-weighted images. The thickness of the articular cartilage of the medial condyle (**a**) and that of the lateral condyle (**b**) are shown

No significant differences were noted in the thickness of the articular cartilage of the medial and lateral condyles of the femur between the control, 2-w OA and 4-w OA (Fig. 4).

### Comparison of histological findings

**Fig. 5:**
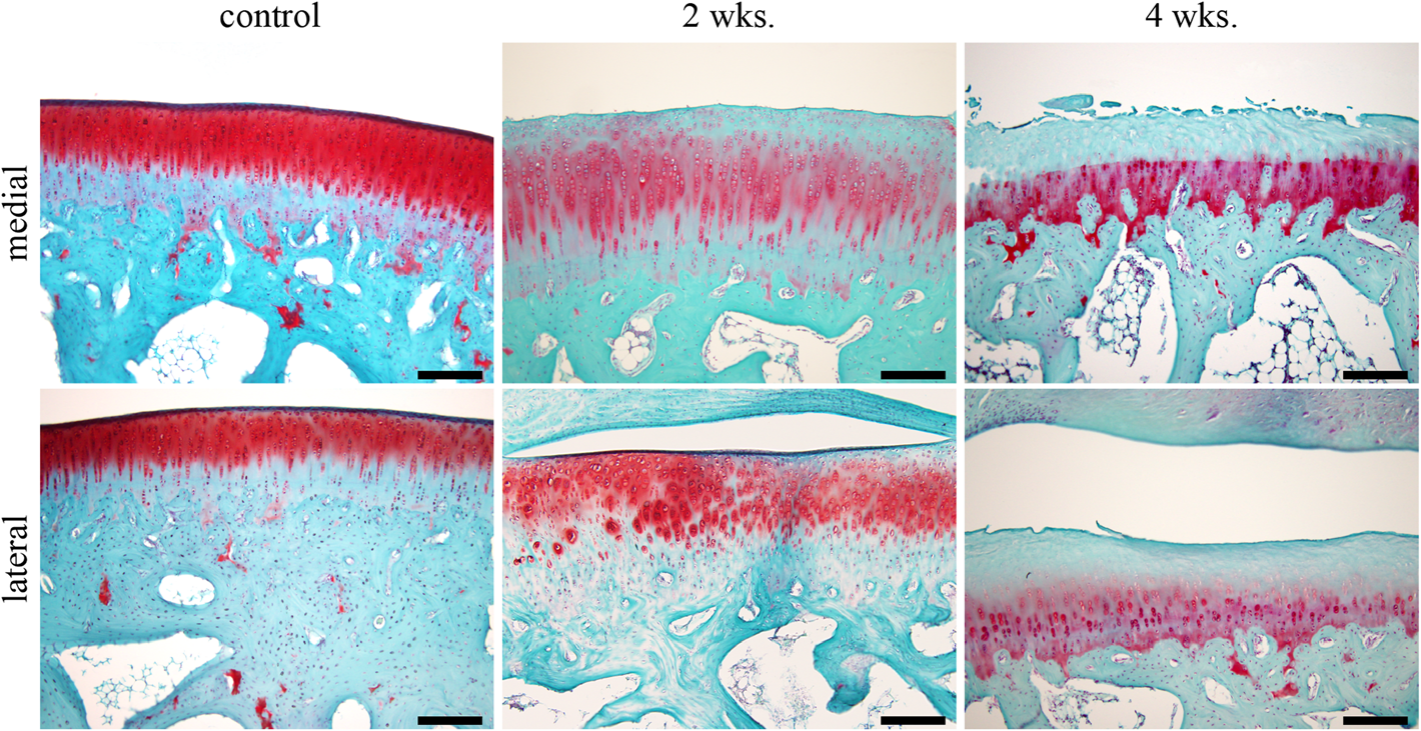
Articular cartilage of the rabbit knee with Safranin O staining. Histological images with Safranin O staining of the medial and lateral condyle of the femur are shown. The top row shows the images of the medial condyle of the control, the 2-w OA and 4-w OA, and the bottom row of the lateral condyle. Scale bar, 200 μm

**Fig. 6:**
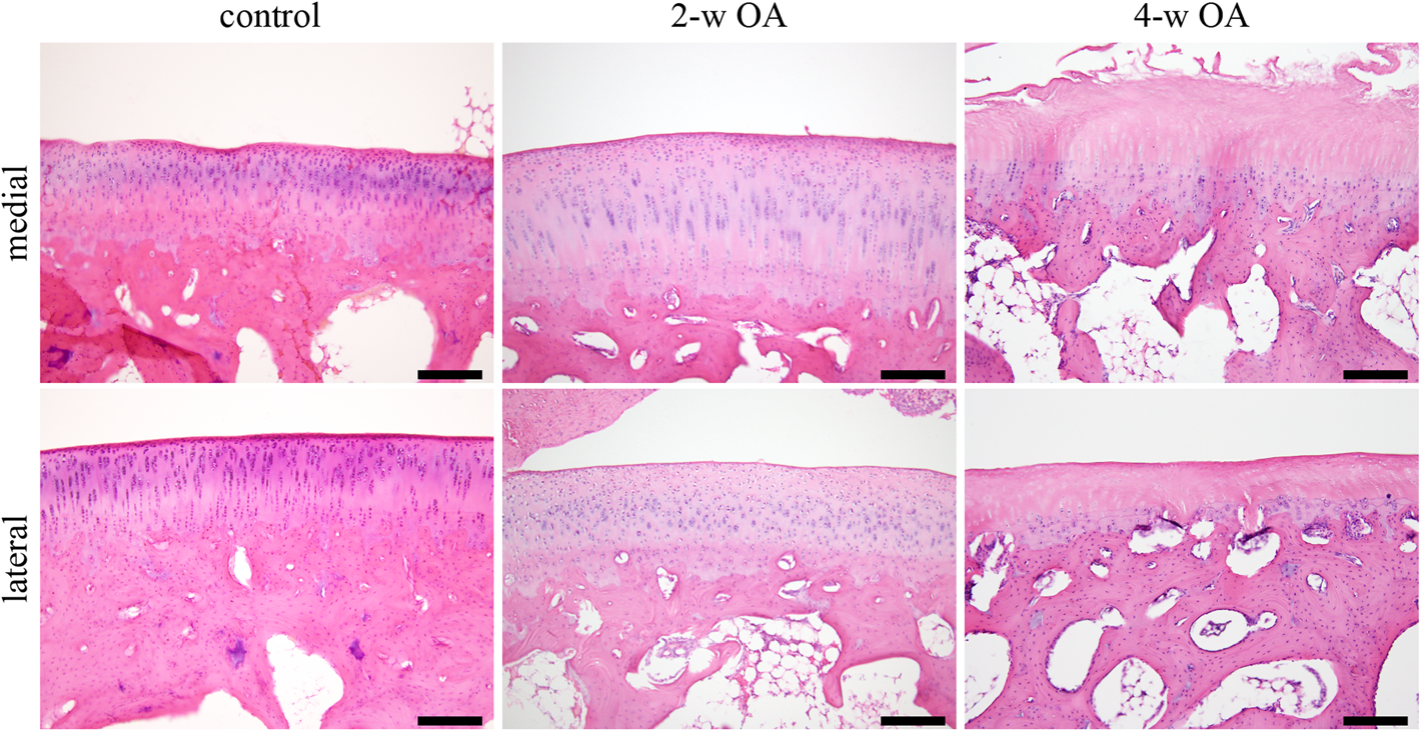
Articular cartilage of the rabbit knee with H&E staining. Histological images with H&E staining of the medial and lateral condyle of the femur are shown. The top row shows the images of the medial condyle of the control, the 2-w OA and 4-w OA, and the bottom row of the lateral condyle. Scale bar, 200 μm

We examined the four evaluable rabbits from the 4-w OA, one rabbit from the 2-w OA and one rabbit from the control. The loss of Safranin O staining and the erosion of the cartilage in the 4-w OA increased compared with the 2-w and control (Fig. 5). For H&E staining, the control exhibited normal findings, and the 2-w OA and 4-w OA exhibited decreased chondral cells (Fig. 6). The Mankin score and the OARSI score were used for the histological evaluation of OA (Table 3). The scores sequentially increased from the control to the 4-w OA.

**Table 3:** Histological evaluation using Mankin score and OARSI score for the 2-w OA and 4-w OA Models

	Mankin score						OARSI score				
3 slices per rabbit		Structure	Cells	Safranin-O staining	Tidemark	Total	Safranin-O staining	Structure	Chondrocyte density	Cluster formation	Total
control (*n* = 1)	medial	0.0 ± 0.0	0.0 ± 0.0	0.0 ± 0.0	0.0 ± 0.0	0.0 ± 0.0	0.0 ± 0.0	0.0 ± 0.0	0.0 ± 0.0	0.0 ± 0.0	0.0 ± 0.0
	lateral	0.0 ± 0.0	0.0 ± 0.0	0.0 ± 0.0	0.0 ± 0.0	0.0 ± 0.0	0.0 ± 0.0	0.0 ± 0.0	0.0 ± 0.0	0.0 ± 0.0	0.0 ± 0.0
2-w OA (*n* = 1)	medial	0.0 ± 0.0	2.0 ± 0.0	1.0 ± 0.0	0.0 ± 0.0	3.0 ± 0.0	1.0 ± 0.0	0.0 ± 0.0	1.0 ± 0.0	1.0 ± 0.0	3.0 ± 0.0
	lateral	0.3 ± 0.6	2.0 ± 0.0	1.7 ± 0.6	0.0 ± 0.0	4.0 ± 1.0	2.3 ± 1.2	1.0 ± 1.7	2.0 ± 0.0	2.3 ± 1.2	7.7 ± 1.5
4-w OA (*n* = 4)	medial	3.0 ± 0.6	3.0 ± 0.0	2.6 ± 0.5	0.1 ± 0.3	8.7 ± 0.8	5.1 ± 1.0	4.9 ± 0.8	4.0 ± 0.0	1.0 ± 0.7	15.0 ± 1.5
	lateral	3.0 ± 0.9	3.0 ± 0.0	2.8 ± 0.4	0.1 ± 0.3	8.9 ± 1.1	5.5 ± 0.8	5.2 ± 0.3	4.0 ± 0.0	1.8 ± 0.6	16.4 ± 1.7

## Discussion

### Assessment of this study

In this study, histological findings revealed substantial changes at 4 weeks after surgery and minimal changes at 2 weeks after surgery. The SIR values of the 2-w OA, 4-w OA and the control after the agent injection were significantly greater than those before the injection. This indicated that selective visualization of the articular cartilage using MRI with this contrast agent could provide a strong contrast between the articular cartilage and joint fluid. This feature allowed the delineation of thin structures, such as rabbit articular cartilage.

The 4-w OA exhibited no significant differences in the thickness of the articular cartilage. The SIR and T_2_ values were significantly different between the control and the 4-w OA only after contrast agent injection. Therefore, MRI with contrast agent enabled us to detect articular cartilage degeneration before we could detect it using conventional MRI. These findings were consistent with the histological findings.

In histological assessment, the lateral condyle indicated more progressive degeneration in articular cartilage. This might relate to the result that the T_2_ values of the lateral condyle in the 4-w OA were significantly greater after contrast agent injection than before the injection.

### Other studies on articular cartilage

T_1ρ_-calculated images, T_2_-calculated images, dGEMRIC, gag CEST, and ^23^Na MRI have been investigated as MRI methods that can potentially be used to evaluate degenerative changes in articular cartilage [9–11]. Li X et al. reported increased T_1ρ_ and T_2_ relaxation times of patellofemoral joint articular cartilage in OA patients compared to those in normal individuals [9]. Rakhra KS et al. measured the T_1ρ_ relaxation time of hip articular cartilage of femoroacetabular impingement patients and normal control individuals [26]. The values were increased in middle and deep layers in the femoroacetabular impingement patients compared to those in the control patients, indicating cartilage changes. Despite these results, the histological evaluation of OA has been difficult to perform in humans. Wei et al. measured the T_2_ relaxation time of an OA model generated by immobilization of the rabbit knee, which exhibited increased values 2 weeks after immobilization and reduced Safranin O staining and PG content [27]. Evaluation by histology and by MRI findings requires an animal model, such as a rabbit. However, the articular cartilage of experimental animals such as rabbits is thinner than that of humans, and the articular cartilage of the femorotibial joint is too thin for clear delineation. The researchers performed qualitative MRI of the articular cartilage of animals [9–11, 26]; however, few studies have delineated the articular cartilage vividly and evaluated it qualitatively [12]. Therefore, methods have not been established to detect earlier stages of OA.

### Characteristics of MRI with the double-contrast agent

Special conditions are needed to clearly visualize thin structures, such as articular cartilage. Wesbey et al. used oral iron solutions to enhance the signal of the gastrointestinal tract of rats and humans during MRI [26]. This method could vividly delineate the gastrointestinal tract because the signal intensity of the cavity was decreased. Similarly, in the present study, the double-contrast agent increased the signal intensity of the articular cartilage and reduced the signal intensity of the joint fluid, which enabled more vivid delineation of the articular cartilage than that achieved by conventional imaging. The SIR values were increased in more progressive OA. This method was effective not only for visualizing articular cartilage but also for detecting OA before articular cartilage degeneration could be detected via conventional MRI.

The contrast agent contains manganese and ferrous ions, which shorten T_1_ and T_2_ relaxation times [20–22, 28]. FeCl_3_ is colloidal; therefore, it cannot infiltrate into the normal cartilage and remains in the joint fluid. The ferrous ions decreased the T_2_ values of the joint fluid, which decreased the signal intensity of the joint fluid, resulting in an increased SIR [23]. The agent contains 0.5 mmol/L MnCl_2_ and 2.5 mmol/L CuSO_4_. The previous study showed that the copper ions have T_1_ shortening effect, though the manganese ions dominated the effect of these ions [23].

Articular cartilage consists of a matrix, including PGs and type II collagen, which has a low T_2_ value, and free water, which has a high T_2_ value [29]. The irregularity and the fissure of degenerative cartilage allowed more manganese ions to enter the cartilage. The T_2_ relaxation time of the matrix with manganese ions was reduced, possibly to less than 11.55 ms, which was the TE of the T_2_-calculated images; therefore, the signal of the matrix was small enough to be negligible. Moreover, an increase in the relative water content caused the increase in T_2_ relaxation time [29]. These reasons may explain why the T_2_ values of cartilage in the 4-w OA were significantly greater than those in the control after contrast agent injection. To explain our results more completely, the mechanism should be ascertained using a multi-exponential analysis of T_2_ times [30].

The significant difference in the SIR values between the control and the 4-w OA was thought to be caused by the increased signal intensity of cartilage. The increase in T_2_ values of degenerative cartilage contributed to the increased signal intensity of cartilage. Additionally, T_1_ relaxation time of free water in cartilage was 3 s. Manganese ions shortened T_1_ relaxation time of water, which strengthened the signal intensity of cartilage in the MR images, with a TR of 2000 ms.

### Limitations

This study had several limitations, one of which is toxicity. Specifically, neurotoxicity has been noted when manganese ions are used in contrast agents [31]. However, abnormal functions and respiratory rates were not observed in this study. Toxicity should be assessed in future studies via cell culture in vitro and animal experiments. The contrast agent contains ferrous ions as negative contrast agents; therefore, low levels of manganese ions were required to obtain sufficient contrast. Inflammatory findings were not observed in this study, which may indicate the safety of this procedure. Another limitation of the study was that only one rabbit from the control group and one from the 2-w OA were assessed histologically.

## Conclusions

We evaluated T_2_ values and SIR values of the knees in a rabbit OA model and in a control model using a new contrast agent. The OA model had significantly higher values for these parameters than the control model after intra-articular injection of contrast agent. MRI with the double-contrast agent enabled us to detect OA earlier than using conventional MRI.

## Abbreviations

dGEMRIC: Delayed gadolinium-enhanced MRI
gag CEST: Glycosaminoglycan chemical exchange saturation transfer
H&E: Hematoxylin and eosin
MRI: Magnetic resonance imaging
OA: Osteoarthritis
OARSI: Osteoarthritis Research Society International
PG: Proteoglycan
ROI: Regions of interest
SIR: Signal intensity ratio
